# Food Safety: Adding Up to No Good?

**Published:** 2006-04

**Authors:** Michael Szpir

The safety of food additives is usually examined by varying the dose of a single additive administered to animal models or cell cultures. However, British researchers at the University of Liverpool and the University of Ulster report in the March 2006 issue of *Toxicological Sciences* that combinations of additives can produce neurotoxic effects at dosages that are safe when each additive is tested alone.

The authors examined four common food additives: Quinoline Yellow (FD&C Yellow No. 10), Brilliant Blue (FD&C Blue No. 1), L-glutamate (the major constituent of monosodium glutamate, or MSG), and aspartame. Quinoline Yellow is banned from foods in the United States, Japan, and Norway; Brilliant Blue was banned from foods in most European countries but has since been reapproved. Coauthor Karen Lau, a doctoral student, says these additives were tested because they are commonly used in foods targeted for consumption by children.

Neurotoxicity was measured by the relative growth of neurites from mouse NB2a neuroblastoma cells after exposure to the additives. Two combinations of additives stunted neurite growth: Quinoline Yellow paired with aspartame, and Brilliant Blue paired with L-glutamatic acid. Other pairings showed no effect. Lau hypothesizes that ingestion of the well-established neurotoxicants aspartic acid and L-glutamic acid as additives could lead to a high enough body burden to kill neurons by a mechanism called excitotoxicity.

Lau says young children may be especially at risk for the type of toxicity observed in the nerve-cell cultures, because effects were seen at concentrations of additives she says are theoretically achievable in plasma by eating foods and drinks typically consumed by children—for example, a snack of corn chips, which may contain MSG, and a fruit juice drink, which may contain aspartame.

Scientists at the U.K. Food Standards Agency (FSA) question whether these results are relevant to the human consumption of these additives. “[The Lau study assumes] that both MSG and aspartame are absorbed one hundred percent in the gut, but [other studies] show that this does not seem to be the case,” says FSA senior press officer Shaun Whelan. “It is . . . extremely unlikely that the plasma levels predicted by the authors of this study accurately reflect the actual situations *in vivo.*”

Whelan says glutamic acid and aspartic acid occur naturally in many foods, and there is no evidence that they are treated differently in the body when they are ingested as food additives. Lau counters, however, that consumption of glutamic acid in its free form or as MSG has a more dramatic effect on plasma levels than that of glutamatic acid in protein, and can lead to high concentrations in the body. More research is needed to clarify these effects.

Other studies have suggested that non-nutritive food additives are associated with behavioral disorders such as attention deficit/hyperactivity disorder. These effects are controversial, but Lau’s team believes their results warrant further investigation of such possibilities. Whelan says the FSA is currently funding research on the effects of ingested chemical mixtures, including color additives, on the behavior of young children.

## Figures and Tables

**Figure:**
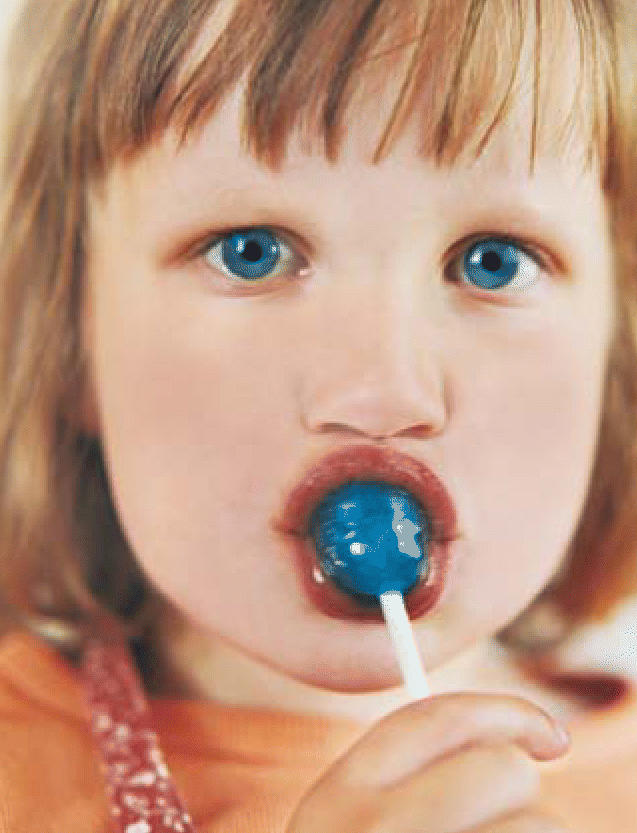
Foodblues? Certain food additives including common dyes may combine to cause toxic effects.

